# Neuroprotective effects of Apigenin on prenatal Valproic acid-induced autism spectrum disorder in rats

**DOI:** 10.1016/j.ibneur.2024.10.003

**Published:** 2024-10-30

**Authors:** Mitra Farbin, Anahita Hejazi, Nahid Fakhraei, Yaser Azizi, Soraya Mehrabi, Razieh Hajisoltani

**Affiliations:** a Physiology Research Center, Iran University of Medical Sciences, Tehran, Iran; b Physiology Department, Iran University of Medical Sciences, Tehran, Iran; c Electrophysiology Research Center, Neuroscience Institute, Tehran University of Medical Sciences, Tehran, Iran; d Department of Neuroscience, Faculty of Advanced Technologies in Medicine, Iran University of Medical Science, Tehran, Iran

**Keywords:** Autism spectrum disorder, Valproic acid, Apigenin, Oxidative stress, Inflammation, Rats

## Abstract

Valproic acid (VPA) demonstrates teratogenic effects during pregnancy. Prenatal exposure to VPA may result in autism spectrum disorder (ASD) -like phenotypes. Apigenin, a natural flavonoid, has been shown to have neuroprotective impacts due to its antioxidant properties. This study aimed to investigate the protective effects of apigenin in prenatal Valproic acid-induced autism in rats. Female rats (220–240 g, 2–3 months) received a single dose of VPA (600 mg/kg, i.p.) on the 12.5th day of gestational. The male offspring were given oral apigenin (50 mg/kg, p.o.) or the vehicle for 30 days. Behavioral tests, biochemical assessments for oxidative stress markers and pro-inflammatory cytokines were performed. VPA-treated rats exhibited increased anxiety-like behavior, and repetitive behavior. Social interaction was reduced, and detection of the novel object was impaired. Also, VPA-treated rats have shown higher levels of oxidative stress malondialdehyde (MDA) and lower GPX and superoxide dismutase (SOD) levels. Furthermore, IL-6 and TNF-α increased in the prefrotalcortex decreased. On the other hand, apigenin-treated rats restored the cognitive consequences and lowered oxidative stress and inflammation in the prefrotalcortex.

**Conclusion:**

Chronic apigenin treatment restored the behavioral and biochemical abnormalities caused by prenatal VPA exposure.

## Introduction

According to the American Psychiatric Association (APA), autism spectrum disorder (ASD) is the one fast-growing pervasive neurodevelopmental disorder defined by chronic impairment in social communication and interactions, as well as restricted, repetitive patterns of behavior, interest, and activities (American psychiatric association, 2013). Autism is associated with increased repetitive movements, stress, abnormal social behaviors, and learning and memory challenges.

The clinical appearance and severity of these fundamental symptoms vary significantly amongst individuals with ASD despite these shared areas of impairment that characterize ASD as a disease. ASD patients frequently have different levels of intellectual functioning in addition to these mainsymptoms; some have high IQs, while others have severe intellectual disabilities (Gilbert et al., 2017, Rahimi et al., 2023, Nicolini and Fahnestock, 2018a). Furthermore, many individuals with ASD exhibit maladaptive emotional responses. Several studies suggest that some individuals with ASD experience heightened levels of anxiety and impaired emotional learning. Furthermore, patients with ASD typically experience a decrease in overall cognitive function scores, with approximately 75 % of them displaying learning and memory dysfunction (Skladal et al., 2003, Gorman et al., 2015, Minshew et al., 1993). The previous study showed that hippocampus is the one of the major area that impaired in autistic animal. Some studies have reported that prenatal exposure to VPA suppresses long-term potentiation (LTP) in hippocampal slices (James et al., 2009, Palmieri et al., 2010).

Anxiety like behavior is the most common comorbid disorder associated with ASD (Heuer et al., 2008). Several studies have found that the rats prenatal exposed to VPA showed the anxiety in elevated pulse maze and open field (Gottfried et al., 2013, Jafarian et al., 2013). From a neurophysiological and morphological standpoint, these rats exhibit functional changes in the prefrontal cortex and some limbic brain regions compared to control animals. The changes are associated with molecular-level changes, including reduced GABA activity in the central nervous system, decreased levels of glutamate decarboxylase (GAD67) in the cortex and hippocampus, and increased expression of glutamate-N-methyl-d-aspartate (NMDA) receptor subunits, vesicular glutamate transporter 1, and oxytocin and its receptor (Frye et al., 2013, Morakotsriwan et al., 2016, Al-Amin et al., 2015, Banerjee et al., 2013).

Animal models are frequently used to investigate the pathological mechanisms of diseases and suggest potential treatments for biological functions. Curiously, findings show that prenatal exposure to Valproic acid (VPA) can induce neurobehavioral abnormalities in rodents, similar to those seen in autism patients. VPA administration during prenatal development has become a widely used animal model to cause autism-like symptoms over recent years (Kumar and Sharma, 2016). This model shows similarities to autistic children regarding impairment in the cranial nerve motor nuclei in the brainstem and reduced Purkinje cells in the cerebellum (Miyazaki et al., 2005). Valproic acid (VPA) has been used to treat epilepsy, bipolar disorder, and migraines by acting as neurotransmitter synthesis and release, immune activation, mitochondrial function, lipid metabolism, and gene expression (Nicolini and Fahnestock, 2018b). Animal studies have demonstrated that exposure to VPA in the prenatal period can cause increased oxidative damage, hyperserotonemia, excitatory/inhibitory imbalance, and cognitive defects (Ahn et al., 2014, De Theije et al., 2014, Sandhya et al., 2012, JK and AM, 2006). In addition, the retrospective and prospective clinical studies have shown that children prenatally exposed to VPA show symptoms of ASD, such as social impairment, communication deficits, anxiety, obsessive/repetitive behavior, cognitive disorders, and motor skills impairment (Li et al., 2009). The model was developed at different embryonic (E) days based on the induction period, but using E-12.5th day for VPA exposure in rats is recommended (Petrelli et al., 2016). After the closure of the neural tube, cranial nerves begin to develop from the 12th to the 13th day of embryonic development, marking a critical period for the formation of sensory and motor nerves (Markakis et al., 2004). Therefore, the offspring of rats treated with VPA on gestational day 12.5 exhibited behaviors, biochemical profiles, and neuroanatomical features similar to those of autistic patients. The impaired social behavior, anxiety, locomotors, and repetitive behavior, like hyperactivity in rats, show similarities to autistic subjects (Popović et al., 2014, Han et al., 2012a, Zhao et al., 2011, Wang et al., 2001). Several studies suggest that genetic factors, immune system disorders, and oxidative stress, as indicated by high levels of lipid peroxidation, are involved in the pathophysiology of individuals with ASD (Losi et al., 2004, Singh et al., 2021). The previous study revealed that oxidative damage can result in physiological abnormalities across various regions associated with speech and auditory processing, memory, social behavior, and anxiety (Li et al., 2015). A low level of reduced glutathione (GSH) has been found in the cerebellum and temporal cortex of autistic patients compared to normal subjects. Furthermore, other markers of oxidative stress, such as malondialdehyde (MDA) and lipid peroxide (LOOH), are elevated in autistic children. Furthermore, high concentrations of several pro-inflammatory cytokines, including interleukin-1 (IL-1), interleukin-6 (IL-6), and tumor necrosis factor-alpha (TNF-α), have been detected in the post-mortem blood and brain cerebral tissue of autistic individuals (Nicolini and Fahnestock, 2018a, Skladal et al., 2003). Given the association of this disease with oxidative damage to the CNS, researchers have recently focused on using antioxidants, especially those present in medicinal plants.

Apigenin (4, 5, 7-trihydroxyflavone), one of the most common flavonoids, has been thought to have a variety of biological functions including antioxidant, anti-inflammatory, neurogenic, neuroprotective, and antitumor (Kohara et al., 2014). Apigenin can pass the blood-brain barrier (BBB) which is critical for the treatment of central nervous system (CNS) disorders (Komaki et al., 2015). Apigenin is known to have anxiolytic and sedative properties. Recent research has confirmed that apigenin has the ability to relax muscles in animal models (Komatsu et al., 2008). Some studies demonstrated that apigenin attenuated anxiety-like behavior by reducing the anxiety index and increasing the time spent in open arms as well as the number of entries into open arms (Morakotsriwan et al., 2016). Apigenin has anxiolytic effect through affected the α-adrenergic, dopaminergic, serotonergic receptors (Gholamzadeh et al., 2021). Furthermore, it has been demonstrated that Apigenin induces anti-anxiety effects by inhibiting the gamma-aminobutyric acid (GABA) and N-methyl-D-aspartate (NMDA) receptors and antagonizing them (Kim et al., 2011, Güldenpfennig et al., 2011, Zhang et al., 2015). Apigenin protects neurons of the hippocampus against neurotoxicity in the kainite model by reducing oxidative stress (Chauhan et al., 2012). Additionally, apigenin has exhibited a neuroprotective effect against the neurotoxicity of amyloid-β peptides (Aβ) in Alzheimer’s disease (AD), controlling redox imbalance and enhancing barrier properties of brain capillary endothelial cells in rats (Lucchina and Depino, 2014). Apigenin enhances antioxidant systems, increasing glutathione peroxidase and reducing oxidative injuries and lipid peroxidation. It also has anti-inflammatory effects by decreasing IL-1β and TNF-α levels (Vig and Jedrysek, 1999, Hegazy et al., 2015).

The present study aimed to investigate the effects of apigenin on VPA-induced autistic behaviors in rats. We wanted to understand whether apigenin's anti-autistic actions are related to changes in oxidative stress marker and pro-inflammatory cytokines. To do this, we examined the effects of apigenin treatment on the production of TNF-α, IL-6, and the levels of SOD, GPx, and MDA expression in the prefrontal cortex (PFC). The PFC is a specific brain region implicated in the pathophysiology of ASD.

## Materials and methods

### Animals

After determining the estrus stage of the breeding cycle, male and female Wistar rats (200–300 g) were mated. Once spermatozoa were found on the vaginal smear, it was designated as the gestation day (GD) “zero. Female rats were housed separately after copulation and kept at a constant temperature (23 ± 3 ˚C) and humidity (30–35 %) under conventional lighting (12:12 h light-dark) with access to food and water ad libitum. The experiment was approved by the institutional ethics committee of Iran University of Medical Sciences (1400–2–32–21).

### Chemicals

Sodium valproate (NaVPA, Sigma, UK), apigenin (Aktin Chemical (China)), carboxymethyl cellulose ((CMC) chemicenter)

### Experimental design

Female pregnant rats (n = 6) were randomly divided into the saline and Valproic acid (VPA) groups. The VPA group received a single intraperitoneal injection of sodium valproate (600 mg/kg, i.p.) on gestational day (GD) 12.5. Sodium valproate (NaVPA) was dissolved in normal saline (Wu et al., 2017). Control females received a single injection of physiological saline at the same time. The offspring received apigenin on days 22–52.

The rats were permitted to raise their own litters and pups until postnatal day (PND) 21 when they were weaned. After weaning, 24 male offspring were housed individually in groups of 3–6 per cage. Then they were divided into three groups of 8 as the following: 1) VPA group: received (i.p) administration of VPA (600 mg/kg on the GD12.5). 2) Control group: received saline i.p. on the GD12.5. 3) Treatment group (VPA + apigenin): received orally apigenin 50 mg/kg/day for 30 days via gavage. The dose of apigenin was chosen based on the previous study, since they found beneficial effects of apigenin in a model of epilepsy (Avallone et al., 2000).

4) Solvent group: received Carboxymethyl cellulose (CMC) orally at a dose of 5 mg/kg/day for 30 days through a gavage.

### Behavioral tests

Behavioral tests were performed on postnatal day 52–60 (PND) in the following section:

### Social interaction

Autism is characterized by difficulties in social interaction. On PND 53, in the present study, a three-chamber social behavior apparatus (114 × 51 × 51 cm) was used to evaluate social interaction. Before beginning the first phase, the rat was acclimated for 5 minutes in the central chamber to enhance rat exploration of the side compartments compared to the center chamber. The first phase, the sociability phase, began after the habituation time of 5 minutes had ended. In the sociability phase, an age and sex-matched stranger rat was put in a small wire cage with a radius of 5.5 cm in either the left or the right chamber. During the sociability phase, one side of the chamber wire cage remained empty. The sociability phase was done for 10 min. Following the completion of the first phase, the second phase began and lasted for 10 minutes. In this phase, a new rat from another control litter was put in the empty chamber under the wire cage, age and sex-matched. During this time, the unfamiliar chamber was called the familiar chamber, while the empty chamber was called the novel chamber. Sociability was defined as "the sociability index (SI), which was described as the ratio of time spent by the test animal on the stranger side to time spent on the empty side." On the other hand, social preference was measured using the social preference index (SPI), which has been described as the ratio of time spent by the test animal on the new vs familiar side (Alghamdi et al., 2022).

### The elevated plus maze (EPM)

Anxiety-like behavior is common in autistic patients, and this behavioral change has been described in VPN-induced ASD. On PND 40, the anxiety behavior was evaluated by the elevated plus maze (EPM). The EPM consisted of two opposite open arms (50 × 10 cm2), enclosed by 40-cm high walls. The maze was elevated 50 cm above the floor. During the 5-minute test period, the time spent in the open and the closed arms was recorded using the video tracking system (Ethovision XT, Noldus, Netherlands). The anxiety index (AI) was calculated as the ratio of time spent in open arms to total times spent in open and closed arms (Gąssowska-Dobrowolska et al., 2020).

### Marble test

Rats were habituated individually in a cage filled with 4 cm of fresh bedding for 10 minutes. Following habituation, rats were taken, and twenty black marbles were put at equal distances in a 4 × 5 arrangement. Two observers recorded more than 50 % of marbles buried with bedding after the 10-minute testing session. After each testing period, the marbles were cleaned with 70 % ethanol (Sharma et al., 2020).

### Novel object recognition test (NOR)

The rat was placed in the white plastic container (75 × 75 × 45 cm) the day before the task. This test is separated into three stages: the habituation stage, the familiarization stage, and the test stage. Each trial was 10 minutes. Within the habitation phase, the rodent could explore the field without objects. Within the acquisition phase, rats were placed within the area and permitted to investigate two identical test objects (A, B), corner to corner each other at a distance of approximately 10 cm. One hour afterward, the exploratory behaviors were tested for 10 minutes as test sessions with one of the objects substituted with a different-shaped object. All trails were recorded and assessed using the Ethovision video tracking system (Ethovision XT, Noldus, Netherlands), and the discrimination index was determined as the ratio of exploratory contacts of a novel object to exploratory contacts of a familiar object (Dourado et al., 2020).

### Biochemical assays

Animals were anesthetized with inhalation of ether, and sacrificed by decapitation using a guillotine on PND 60, and the prefrotalcortex were extracted and homogenized in phosphate buffer (pH 7.4) using Teflon Homogenize. The homogenate was centrifuged at 3000 rpm for 15 minutes. The homogenate's supernatant was separated for several biochemical estimates using the procedures listed below:

### Oxidative stress

It has been suggested that oxidative stress plays a role in the etiology of autism (Gąssowska-Dobrowolska et al., 2020). As oxidative stress markers, prefrontal cortex superoxide dismutase (SOD), glutathione levels (GPX), and malondialdehyde (MDA) were measured in this study. After defrizzing the prefrontal cortex tissues, the samples containing cold PBS (1 mL/100 mg tissue) were homogenized and centrifuged at four °C at 14,000 rpm/min (5 min), and the supernatant was then obtained. Commercial kits were purchased from Nvand Salamat, Uremia, Iran.

### Malondialdehyde (MDA)

As previously described, lipid peroxidation was measured colorimetrically using thiobarbituric acid reactive substances (TBARS). The samples (100 M) were loaded into fresh tubes, and then acetic acid, sodium hydroxide, and thiobarbituric acid (TBA) were added before the tubes were immersed in boiling water (60 min). Because MDA reacts with TBA to generate a purple hue, the samples were placed on ice for 10 minutes to stop the dyeing process. A microplate reader was used to measure the absorbance at 540 nm. Using a standard curve, the concentration of MDA was determined and expressed as mol/mg of tissue protein (Fallah et al., 2019).

### Superoxide dismutase (SOD) activity

SOD is the most powerful enzymatic antioxidant protection system in cells. SOD catalyzed superoxide ions (O2• -) to oxygen and H2O2. Working solution (assay buffer + tetrazolium salt + xanthine oxidase), dilution buffer, and enzyme working solution were added to samples, which were then placed in a 37°C incubator (20 min). By dismuting O2- to O2 and H2O2, SOD presence in samples prevented the conversion of tetrazolium to colorful formazan. A microplate reader was used to measure the absorbance at 460 nm. Using a standard curve, SOD activity was calculated as U/mg of tissue protein (Patel and Singh, 2022).

### Glutathione levels (GP_X_)

Glutathione peroxidase (GPx) is an enzyme that helps protect the body from oxidative damage. Its main function is to reduce lipid hydroperoxides to their corresponding alcohols and to convert free hydrogen peroxide into water. GPx belongs to a family of enzymes with peroxidase activity and is essential for maintaining the body's overall health. Activity of glutathione peroxidase is measured spectrophotometrically. A widely used direct assay links the peroxidase reaction with glutathione reductase and measures the conversion of NADPH to NADP.

### Inflammation factors

The interleukin-6 (IL-6) and tumor necrosis factor-alpha (TNF-α) in the prefrotalcortex were evaluated using RayBio® and Rat ELISA kits (R&D), respectively (TNF-α: CAT.NUMBER. RTA00, IL-6: CAT. NUMBER. R6000B, IL1-β: CAT.NUMBER. RLB00). All kits use sandwich in-vitro enzyme-linked immunosorbent assays (ELISA) to quantitatively measure IL-6 and TNF-α levels in the supernatants at 450 nm on a 96-well plate reader. The data were given in pg/mg of protein.

### Statistical analysis

All data were presented as mean ± SEM and were analyzed using the commercially available software GraphPad Prism® 6.0 (GraphPad, La Jolla, CA, USA). One-way ANOVA, Two-way ANOVA, and two-tailed unpaired Student’s t-test were used for multiple comparisons followed by the Tukey and the Bonferroni post hoc tests, receptively. A probability of 0.05 was considered as the criterion for significance.

## Results

### Social interaction test

**Fig. 1:**
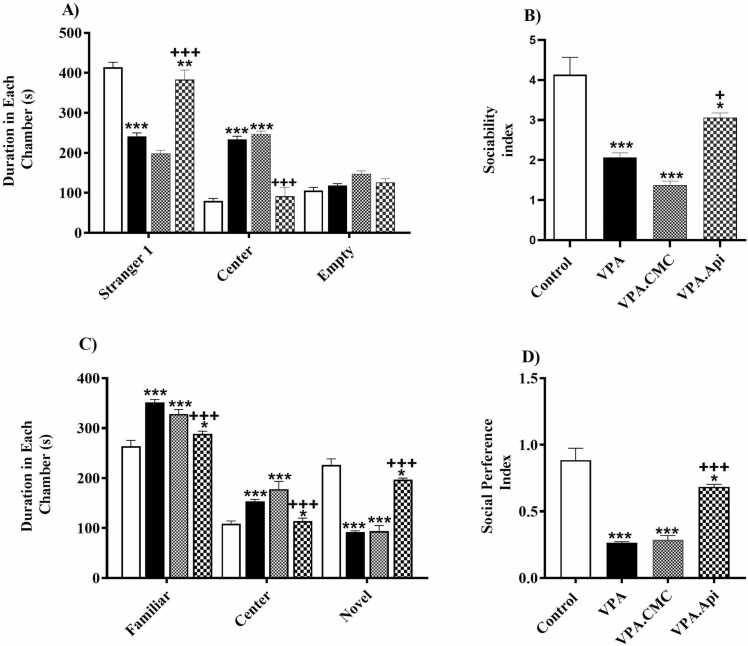
Evaluation of social behavior using three chamber apparatus A) Two-way ANOVA analysis showed that prenatal exposed to VPA caused social deficits, as demonstrated significantly less time spent with stranger 1 and more time spent in the center chamber when compared to control rats. B) Treatment, One ANOVA revealed a significant reduce in the sociability index, as the ratio of time spent in stranger 1 side over empty side. C) On day 4, VPA-treated rat spent more time both with the familiar rat and in the center compartment, but less time with the novel rat. D) One ANOVA revealed a significant reduction in the preference index, as the ratio of time spent in novel side over the time spent in familiar side. Data represented as the mean ± SEM (n = 8, males). *p<0.05, **p<0.01, ***p<0.001 when compared to control. +p<0.05, ++p<0.01, +++p<0.001 when compared to VPA-treated rat.

At first, we evaluated whether ASD-like social interaction deficits in VPA-treated rats would be ameliorated by chronic apigenin administration. Our findings showed that there was a significant decrease in the time spent with the stranger one (241.6 ± 8.296 vs 413.8 ± 12.81, respectively, P < 0.0001) and an increase in the time spent in the central chamber as compared to the control rats (233.8 ± 7.556 vs 80.13 ± 5.935, P < 0.0001). Chronic administration of apigenin improved the deficiencies in social interaction in VPA-treated rats. Our results showed that apigenin treatment ameliorated VPA-exposed rat induced-reduction of time spent in the side of stranger one [383.1 ± 8.547, F (2,21) = 82.68, P < 0.000] and increase of time spent in the center (91.38 ± 8.078, F (2,21) = 139.8, P < 0.0001), and significantly improved sociability index [F (2,21) = 15.21, P < 0.0001], Fig. 1A & B. Then, the social novelty recognition was evaluated by placing a second stranger rat (novel) in the cage which was still empty. VPA-treated rats spent less time in the novel chamber than the familiar chamber compared with the control rats, showing typical social preference behavior (92.25 ± 2.305 vs 226.6 ± 12.03, respectively, P < 0.0001, higher in familiar chamber 351.4 ± 6.065 vs 263.9 ± 11.71). Treatment with apigenin restored the social novelty recognition in VPA-treated rats. Apigenin + VPA rats spent more time in the novel chamber [197.1 ± 2.979, F (2,21) = 94.17, P < 0.0001], and less time in the familiar chamber [288.6 ± 5.352, F (2,21) = 30.12, P<0.0001]. These animals also displayed an increase in the social preference index as compared to VPA-treated rats [F (2,21) = 36.54, P < 0.0001], Fig. 1C & D.

### Anxiety-like behavior

**Fig. 2:**
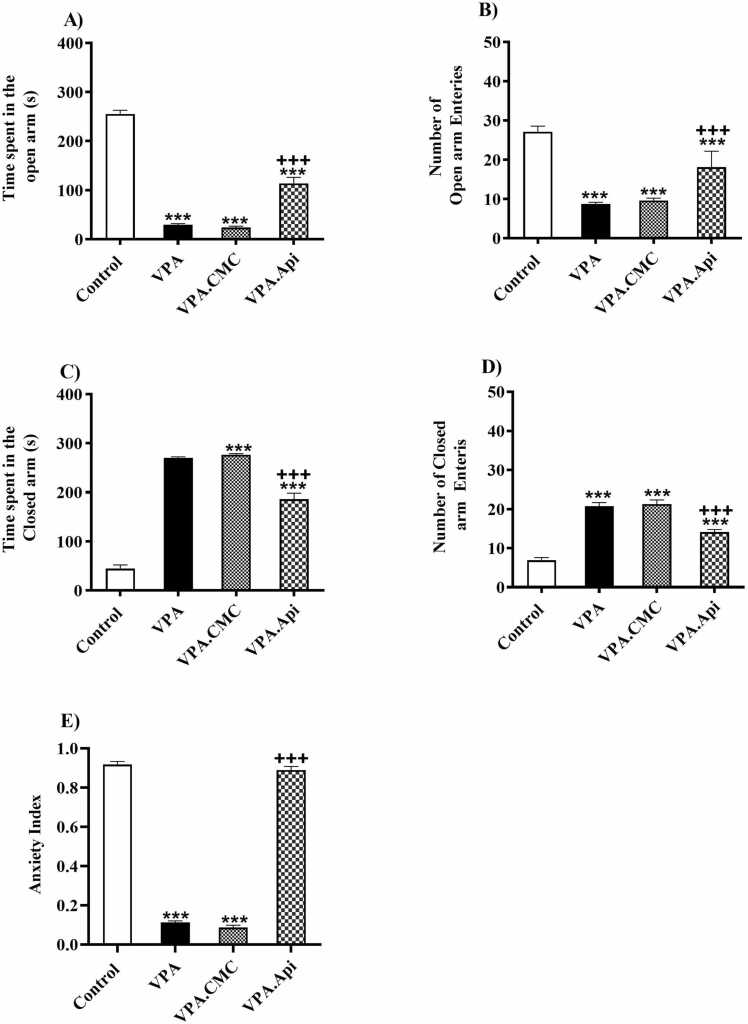
Evaluation of anxiety-related behavior in using the elevated plus maze (EPM). A) VPA exposed rats spent less time in open arms of the elevated plus maze (B). In the EPM test, VPA-exposed rats showed a significant reduced in the anxiety index, which is the ratio of time spent in open to the total time spent in both open and closed arms. Data represented as the mean ± SEM (n = 8, males). *p<0.05, **p<0.01, ***p<0.001 when compared to control. +p<0.05, ++p<0.01, +++p<0.001 when compared to VPA-treated rat.

In the EPM test, control rats compared to VPA-treated rats spent less time in the open arm and number of open arm entries decreased which showed the VPA rats had anxiety-like behavior of rats (255.5 ± 7.457 in VPA group vs 29.75 ± 2.094 in the control group), open arm entries (VPA: 8.750±0.4119 vs control: 27.13± 1.42) but VPA-treated rats spent more time in the closed arm and number of closed arm entries increased (270.3 ± 2.094 in VPA group vs 44.50± 7.45 in the control group), closed arm entries (VPA: 20.75 ±0.88 vs control: 6.87± 0.71).Time spent in the open arm and number of entries was significantly increased following Apigenin administration as compared to VPA-treated rats (113.9 ± 12.22, F (2,21) = 186.6, P < 0.0001, 18.13 ±1.43, F (3,28) = 64.81, P<0.0001, Fig. 2A,B), but time spent and closed arms entries decreased in Apigenin groups(186.1±12.22, F (3,28) = 215.5, P<0.0001) closed arm entries (14.13±0.63, F(3,28) = 64.28, P<0.0001], Fig. 2C,D.group). In addition, the anxiety index in VPA-treated rats markedly decreased compared to control rats (0.11 ± 0.008 in VPA group vs 0.91 ± 0.015 in the control rats (0.88 ± 0.01 in Apigenin + VPA, [F(2,21) = 985.5, P < 0.0001], Fig. 2E. group). Treatment with apigenin significantly improved anxiety index in the VPA-treated.

### Repetitive behavior

**Fig. 3:**
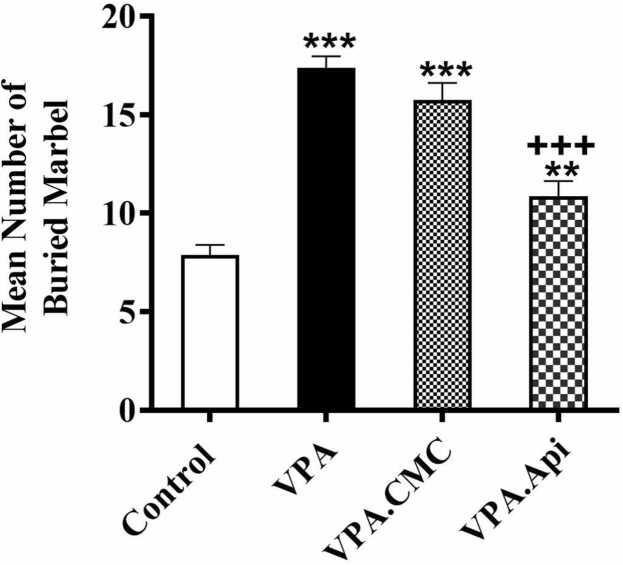
Evaluation of repetitive behavior using the Marbel Burring test. VPA exposure increased the repetitive behaviors in rat. Treatment with apigenin decreased the repetitive behaviors in rat prenatally exposed to VPA. Data represented as the mean ± SEM (n = 8, males). *p<0.05, **p<0.01, ***p<0.001 when compared to control. +p<0.05, ++p<0.01, +++p<0.001 when compared to VPA-treated rats.

To investigate the repetitive behavioral, the marble burying test was performed. The control rats buried fewer marbles than VPA-treated rats (7.87 ± 0.51 in control vs 17.38 ± 0.59 in VPA). Treatment with apigenin markedly decreased marble burying behavior when compared to VPA-treated rats (10.88 ± 0.7662, F(2,21) = 58.59, P < 0.0001, Fig. 3).

### The novel object recognition discrimination

**Fig. 4:**
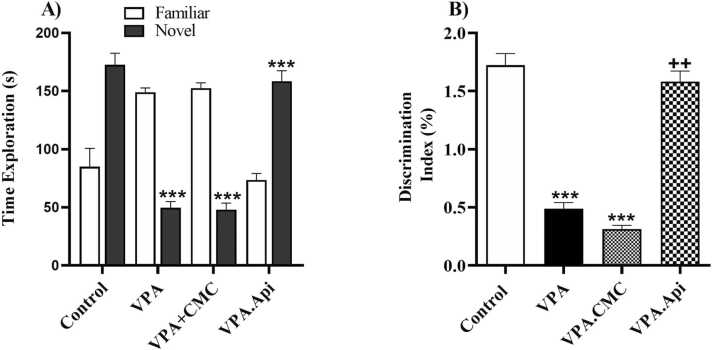
Evaluation of exploratory behavior using novel object recognition test. VPA exposure decreased the exploration behaviors in rat. Treatment with apigenin increased the exploration behaviors in rat prenatally exposed to VPA. Data represented as the mean ± SEM (n = 8, males). *p<0.05, **p<0.01, ***p<0.001 when compared to control. +p<0.05, ++p<0.01, +++p<0.001 when compared to VPA-treated rat.

Time spent with the novel object was significantly decreased as compared with familiar object in the VPA-treated rats (49.63 ± 5.33 exploration time of novel object vs 149.0 ± 3.77 exploration time of familiar object, t-test, P < 0.0001). In addition, novelty detection was evaluated by calculating the discrimination index. The discrimination index significantly decreased in VPA-treated rats when compared to control rats (0.43 ± 0.07 in VPA vs 1.41 ± 0.19 in control, P < 0.001), but treatment with apigenin significantly increased this index [1.26 ± 0.13, F(2,21) = 14.29, P = 0.0001], Fig. 4.

### Oxidative stress

**Fig. 5:**
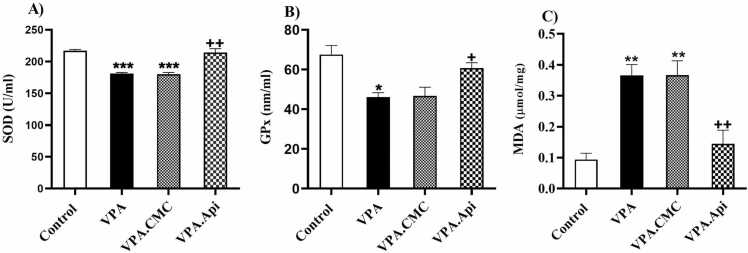
Evaluation of oxidative stress markers between groups (n = 3). A: VPA decreased SOD and treatment with apigenin increased SOD in prefrontal cortex. B: VPA decreased GPx in prefrontal cortex and treatment with apigenin increased GPx. C: VPA decreased MDA and Treatment with apigenin decreased MDA in prefrontal cortex. Data represented as the mean ± SEM (n = 8, males). *p<0.05, **p<0.01, ***p<0.001 when compared to control. +p<0.05, ++p<0.01, +++p<0.001 when compared to VPA-treated rat.

MDA, GPx, and SOD activities were measured in the prefrotalcortex using the calorimetric method (Fig. 5.A-C). Treatment by apigenin significantly reduced MDA level (0.14 ± 0.044, p < 0.01) in the prefrotalcortex whereas increased the GPx (60.74 ± 2.71, p < 0.05), and SOD activity (214.4 ± 5.924, p < 0.001) highlighting the antioxidant effect of apigenin in the prefrotalcortex. Furthermore, our findings showed that prenatal exposed to VPA resulted in oxidative stress damage since in the VPA group, the MDA level was significantly increased (0.36 ± 0.03, p<0.01), whereas GPx (46.05 ± 2.286), and SOD (181.1 ± 1.763) activity was attenuated in comparison with the control group (GPx: 67.54 ± 4.593, SOD: 217.1 ± 2.08). Fig. 5.

### The neuro-inflammation (IL-6, and TNF-α)

**Fig. 6:**
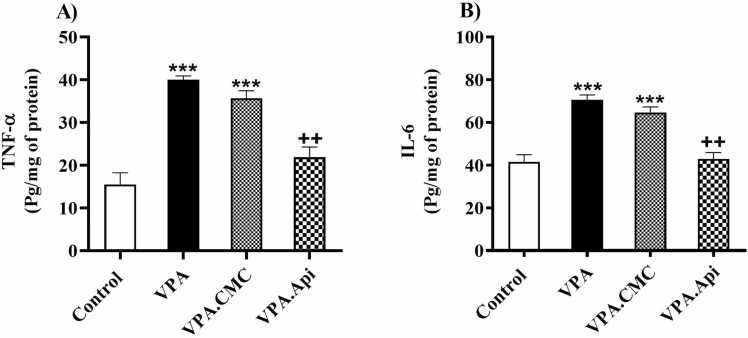
Evaluation of inflammatory markers between groups (n = 3). A: VPA increased IL-1β and treatment with apigenin decreased IL-1β in prefrontal cortex. B: VPA increased TNF-α and treatment with apigenin decreased TNF-α in prefrontal cortex. Data represented as the mean ± SE (n = 8, males). *p<0.05, **p<0.01, ***p<0.001 when compared to control. +p<0.05, ++p<0.01, +++p<0.001 when compared to VPA-treated rat.

VPA treated rats showed a significant elevation in IL-6 (70.65 ± 2.22), and TNF-α levels (40.00 ± 0.8719) in the prefrotalcortex. These results showed that VPA-treated rats had more neuro-inflammation than the control rats. Treatment by apigenin significantly reduced the levels of IL-6 [42.91 ± 3.004, F(2,6) = 31.94, P = 0.0006], Fig. 6A, and TNF-α [21.86 ± 2.37, F(2,6) = 34.97, P = 0.0005], Fig. 6B, in the prefrontal cortex, respectively.

## Discussion

The present study's main findings include 1) Prenatal exposure to VPA-induced autistic-like behavior in their male offspring. 2) Apigenin improved autistic-like behavior. 3) MDA levels increased, while SOD and GPx levels decreased in the prefrontal cortex of VPA-treated rats. 4) Apigenin improved oxidative stress parameters. 5) Inflammatory factors, including IL-6 and TNF-α levels, increased in the prefrontal cortex of VPA-treated rats. 6) Apegenin mitigated inflammatory factors.

Prenatal VPA exposure has been well documented to cause behavioral, biochemical, and pathophysiological impairments in rodents, similar to autistic children. This model is commonly utilized to induce ASD-like symptoms in animal studies. Previous studies have demonstrated that prenatal exposure to VPA leads to various abnormal behaviors, such as a decrease in social interaction, repetitive behavior, exploratory activity, and an increase in motility and anxiety (Han et al., 2012a, Salehi et al., 2019, Han et al., 2012b). Our findings align with these previous studies.

Treatment with apigenin for 30 days significantly improved behavioral outcomes compared to rats exposed to VPA. Apigenin improved social interaction and anxiety-like behaviors. Previous studies have shown that apigenin alleviates depressive-like behavior and anxiety in rat models of depression (Hegazy et al., 2015). Oxidative stress and peripheral and neuronal inflammation may be involved in behavioral alternation in ASD. Investigating the pathways and structures potentially responsible for these behavioral impairments holds promise for further research into the molecular changes associated with ASD.

Oxidative stress, mitochondrial dysfunction, and inflammation have been demonstrated in the brains of rats prenatally exposed to VPA, which can initiate the development of reactive oxygen species (ROS) (Losi et al., 2004). It is well known that brain tissue at the early stage of development is vulnerable to oxidative damage. ROS may impair neurodevelopment by damaging the neuronal cells' lipids, proteins, and DNA. One of the main reasons for autistic symptoms may be elevated nitric oxide and lipid peroxidation levels, which are followed by a change in the oxidative stress markers (Codagnone et al., 2015). Previous studies suggest that oxidative stress, environmental, and genetic factors play a significant role in ASD pathophysiology (Yadav et al., 2022). Increasing levels of pre-oxidative factors like NO, xanthin oxidase(XO), and homocysteine while decreasing antioxidative elements such as ceruloplasmin, transferrin, SOD, GPX, catalase, and glutathione, can lead to oxidative stress (Taha et al., 2023). This Oxidative stress may result in lipid defects in cell membranes, mitochondrial dysfunction, inflammation, and autism-like behaviors in children (Bynagari-Settipalli et al., 2012). According to clinical studies, using antioxidants has improved treating autistic behavior. Prenatal exposure to VPA induces a decrease in GSH, CAT, and SOD and an increase in MDA levels. It has been demonstrated that decreased activities of GSH and SOD are related to lowering neuroprotection against oxidative stress. This suggests that the antioxidative stress system is essential for normalizing these changes and treating autism (Souza et al., 2015). However, Fenofibrate, as an antioxidant, improves these factors and reduces MDA levels in the prefrontal cortex. CAT and GSH are known antioxidant enzymes to play a significant role in the antioxidant enzymes. Therefore, reducing their activity can lead to a decrease in antioxidant capacity (Bynagari-Settipalli et al., 2012, Kalivarathan et al., 2017). Furthermore, Resveratrol, as an antioxidant, reduces MDA and decreases the total GSH, SOD as well as CAT in the brain in a rat model of ASD (Liu et al., 2011). Al-Amin et al. Found Astaxanthin, as an antioxidant agent, improved oxidative stress by increasing GSH, SOD, and CAT and reducing MDA and NO in the prefrontal cortex in the rat prenatal exposure to VPA[64]. Our findings revealed an increase in MDA levels and a decrease in GSH and SOD levels in the prefrontal cortex region of the brain, indicating the induction of oxidative stress and a weakened antioxidative defense. However, apigenin significantly reversed these alterations, which reduced oxidative stress in the rats' prefrontal cortex region. Previous research has shown that apigenin could improve depressive-like behaviors (Miyazaki et al., 2005), as well as reduce oxidative stress and inflammation associated with depression and anxiety disorders (Han et al., 2012a). The antioxidant properties of apigenin have been demonstrated to improve oxidative stress by increasing the activity of antioxidant enzymes such as catalase, superoxide dismutase(SOD), and Glutathione(GSH) [65]. Singh et al. showed that apigenin decreases MDA and increases GSH, SOD, and catalase levels in a rat model of multiple sclerosis [65]. In addition, Oral administration of apigenin reduced glutathione (GSH) levels and significantly decreased MDA and catalase activity in the brain of a rat model of stress (Kim et al., 2011). Kim et al. demonstrated that Apigenin treatment reduced cognitive dysfunction and neuronal damage in an Alzheimer's mouse model by significantly decreasing MDA levels[66]. Given that oxidative stress plays a significant factor in the development of autism, the beneficial effects of apigenin on animal behavior observed in this study may be attributable to its modulation of oxidative stress.

Several studies indicate that individuals with autism may have an immune system dysfunction in the CNS, leading to elevated cytokine levels such as TNF-α, IL1.β, and IL-6 (Kalivarathan et al., 2017). Additionally, studies have shown that prenatal exposure to VPA can increase susceptibility to inflammation in both the peripheral and neuronal systems, resulting in increased expression of inflammation-related genes. Mirza et al. l; demonstrated that prenatal exposure to VPA induces an increase in IL-6, TNF-α, and IL-10 in the prefrontal cortex[67]. Our study revealed increased inflammatory cytokines like TNF-α and IL-6 in rats treated with VPA in the prefrontal cortex, aligning with previous findings demonstrating elevated cytokine levels following prenatal VPA exposure (Kalivarathan et al., 2017, Liu et al., 2011). Chronic inflammation and immune response alterations in the brains of autistic children can lead to cognitive deficiencies [68]. Furthermore, prenatal exposure to VPA has been linked to higher levels of IL-1β, IL-6, and TNF-α, which can result in a decline in cognitive function. Interestingly, high levels of IL-6 and TNF-α in the brain have been associated with anxiety behavior in rodents. It has been demonstrated that treatment with fenofibrate significantly attenuated prenatal VPA-induced anxiety and low exploratory activity by reducing IL-6 and TNF-α in the prefrontal cortex (Kalivarathan et al., 2017). In addition, Bhandari et al. found that resveratrol improved anxiety and cognitive function via decreasing TNF-α levels in the brain in rats treated with VPA (Liu et al., 2011). Furthermore, administering TNF-α neutralizing antibodies to rodents has been found to reduce their anxiety-like behavior. Our findings showed that treatment with apigenin alleviates cognitive impairment by reducing TNF-α and IL-6 levels in VPA-treated rats. Recently, apigenin (20 mg/kg, p.o., daily for 30 days) attenuated microglial activation and restored cognitive function in methotrexate‑treated rats. Apigenin mitigated the MTX-induced neurotoxicity by reversing the biochemical, histopathological, and behavioral derangements tested by novel object recognition and Morris water maze tests. Conclusively, Apigenin lessens MTX-induced neuro-inflammation, oxidative stress, and apoptosis and boosts cognitive function by inhibiting microglial activation [71]. Furthermore, anxiety triggers inflammatory processes by causing high levels of pro-inflammatory cytokines such as IL-6, IL-1β, and TNF-α. This is due to the impact of these cytokines on neurotransmitters associated with the anxiety response. It has been established that oxidative stress and inflammatory-related transcription factors play an important role in the development of anxiety disorders. Apigenin inhibits the production of proinflammatory cytokines, which may be linked to its anxiolytic effect[72].

## Conclusion

In our experiment, prenatal VPA-induced neurotoxicity and cognitive impairment in male offspring could be attributed to the oxidative and neuro-inflammatory responses. This study advocates the neuroprotective effects of apigenin through its lessening neuro-inflammation, oxidative stress, and apoptosis while boosting cognitive function. However, regarding its anti-inflammatory and antioxidative properties, chronic treatment with apigenin markedly reversed these pathological consequences. Our findings suggest that apigenin might be a viable option for attenuating neuro-inflammation and neurotoxicity resulting from prenatal exposure to teratogens, especially VPA.

## Ethical approval

All experimental procedures were carried out in accordance with the guidelines of the Ethic Committee of Iran University of Medical Science, Tehran, Iran. All efforts were made to reduce the number of animals used and their suffering.

## Consent to participate

Not applicable. There isn’t any patient in this research study.

## Consent for publication

Not applicable. There isn’t any patient in this research study.

## Funding

This study was supported by a grant received from the Iran University of Medical Sciences, Tehran, Iran (1400–2–32–21).

## CRediT authorship contribution statement

**Anahita Hejazi:** Investigation. **Mitra farbin:** Investigation. **soraya mehrabi:** Funding acquisition. **yaser azizi:** Formal analysis. **Leila Simani:** Writing – review & editing. **Nahid Fakhraei:** Writing – review & editing. **razieh hajisoltani:** Writing – review & editing, Supervision, Project administration.

## Author contribution statement

Razieh Hajisoltani conceived the original idea and supervised the project. Mitra Farbin,and Anahita Hejazi carried out the experiment, Soraya Mehrabi and Yaser Azizi performed the analytic calculations, Nahid Fakhraei, and Leila Simani help to edit the manuscript. All authors discussed the results and contributed to the final manuscript.

## Declaration of Competing Interest

We have no conflicts of interest to disclose.

## Data Availability

Not applicable
